# Interkingdom Biofilms Are Affected by Non-Antibiotic Strategies: In Vitro Study in Lubbock Chronic Wound Biofilm Model

**DOI:** 10.3390/ijms262311658

**Published:** 2025-12-02

**Authors:** Paola Di Fermo, Firas Diban, Emanuela Di Campli, Luigina Cellini, Morena Pinti, Mara Di Giulio, Morena Petrini, Simonetta D’Ercole, Silvia Di Lodovico

**Affiliations:** 1 Department of Medical, Oral and Biotechnological Sciences, University “G. d’Annunzio” Chieti-Pescara, 66100 Chieti, Italy; paola.difermo@unich.it (P.D.F.); morena.petrini@unich.it (M.P.); 2 Department of Pharmacy, University “G. d’Annunzio” Chieti-Pescara, 66100 Chieti, Italy; firas.diban@unich.it (F.D.); edicampli@unich.it (E.D.C.); l.cellini@unich.it (L.C.); morena.pinti@phd.unich.it (M.P.); mara.digiulio@unich.it (M.D.G.); silvia.dilodovico@unich.it (S.D.L.)

**Keywords:** antimicrobial resistance, interkingdom biofilm, light-emitting diodes, complex magnetic fields, methylglyoxal, Lubbock chronic wound biofilm model, non-antibiotic strategies, photodynamic inactivation

## Abstract

Chronic wound infections associated with resistant polymicrobial biofilms are often refractory to conventional therapies with sustained healing time. This study evaluated the efficacy of non-antibiotic treatments including Methylglyoxal—MGO—Light-Emitting Diode—LED—and Complex Magnetic Fields—CMFs—alone/combined against the biofilms of two polymicrobial mixes (MIX 1, MIX 2) containing *S. aureus*, *P. aeruginosa* and *C. albicans* using the Lubbock chronic wound biofilm model. At 24 h after treatment, the effects were evaluated by (i) CFU/mg reduction, (ii) Confocal Laser Scanning Microscopy—CLSM—and (iii) Scanning Electron Microscopy—SEM. All treatments significantly reduced biofilms in terms of CFU/mg in both mixes versus the controls, 24 h after treatment. MGO showed remarkable activity, especially against *P. aeruginosa*. In MIX 1, LED/MGO + LED was highly effective against *C. albicans*. The combinations MGO + LED/MGO + CMFs enhanced the antibiofilm activity compared to each single treatment against *S. aureus* and *P. aeruginosa*, in both MIX1/MIX2. CLSM and SEM analysis showed biofilm disaggregation and reduction in cell viability with combined treatments, and *Candida* hyphal inhibition after CMFs application. In conclusion, the results demonstrate that MGO, alone or combined with LED or CMFs, shows high efficacy against resistant biofilms in the LCWB model 24 h after treatment, and encourage further studies on potential non-antibiotic and eco-friendly strategies as future alternative therapeutic approaches for chronic wound infections.

## 1. Introduction

Polymicrobial biofilms consist of microbial communities of bacteria, fungi or both, enclosed in a self-produced extracellular matrix that provides protection against external treatments and enhances microbial survival [1]. Biofilms represent a significant clinical challenge due to their extreme tolerance to antimicrobial agents, being up to 10–1000 times more resistant to the antibiotics than their planktonic phase, and microorganisms can escape the host immune system in biofilm [2]. In fact, most infectious diseases are associated with biofilms [3]. The resilience of biofilm-associated infections is particularly problematic in the context of chronic wound infections, where biofilms play a key role in sustaining persistent inflammation, impairing tissue regeneration and delaying wound healing [1,2].

The complex architecture of polymicrobial biofilms promotes cell survival and often includes multidrug-resistant microorganisms such as *Staphylococcus aureus*, *Pseudomonas aeruginosa* and *Candida albicans*, whose complex synergistic interactions further complicate eradication efforts [4]. Among these interactions, *S. aureus* can bind to the hyphal adhesin Als3p of *C. albicans*, facilitating dual-species biofilm formation [5]. Moreover, *C. albicans* secretes the quorum-sensing-related autoinducer signal molecule farnesol and the metabolite prostaglandin E2, which enhances *S. aureus* resistance to vancomycin by upregulating efflux pumps and promoting both planktonic and biofilm growth [6,7]. This complex interaction between microorganisms also includes *P. aeruginosa*. In fact, *P. aeruginosa*, secreting alkyl-quinolones, induces *S. aureus* small-colony variant formation, enhancing its tolerance to antibiotics such as tobramycin [8,9]. The interaction between *P. aeruginosa* and *C. albicans* plays a significant role in disease progression. In a first phase, *P. aeruginosa* exhibits antagonism toward *C. albicans* by inhibiting its growth and yeast-to-hyphae transition [10], and over time, this interaction shifts from antagonism to synergism, and *P. aeruginosa* uses *C. albicans* hyphae as a scaffold for biofilm formation [11].

The spatial organization of microorganisms within chronic wounds reflects these complex interactions. *S. aureus* is most often isolated close to the wound surface, while *P. aeruginosa* colonizes the deeper part of the wound bed, where it maintains a chronic inflammatory state by secreting virulence factors [12,13]. These microbial interactions and the spatial distribution contribute to infections that are often refractory to conventional therapies, increasing patient morbidity, healthcare costs and the risk of complications. Targeting biofilm-associated infections is therefore a critical challenge.

The Lubbock chronic wound biofilm (LCWB) model is a validated in vitro wound infection model that closely mimics both the microenvironment and spatial organization of chronic wound biofilm, including the main pathogens isolated in wound infections, such as *S. aureus*, *P. aeruginosa* and *C. albicans*. In particular, *S. aureus* coagulase activity produces a fibrin network that acts as a scaffold for microbial adhesion [1].

The present study focused on evaluating the efficacy of alternative non-antibiotic strategies, such as Methylglyoxal (MGO), Light-Emitting Diode (LED) irradiation and Complex Magnetic Fields (CMFs), alone and in combination with each other. These approaches aim to overcome antibiotic resistance in chronic wound infections, where conventional therapies often fail. The objective is to identify novel treatments with benefits that include not only a broad-spectrum efficacy but also improved safety and tolerability, environmental sustainability and suitability for short-term applications, to reduce the treatment burden and improve patient compliance. MGO, LED and CMFs were selected based on their ability to meet these criteria. MGO is the active compound responsible for the non-peroxidase antimicrobial activity of Manuka honey (*Leptospermum scoparium*). Manuka honey, due to its high MGO content, has been extensively employed in clinical wound management for its combined antimicrobial, anti-inflammatory and pro-healing effects [14]. LED irradiation is a non-invasive, localized medical treatment that promotes tissue repair and exerts antimicrobial effects through photobiomodulation [15,16,17]. In this study, CMFs generating pulsed electromagnetic fields at extremely low frequencies (1–250 Hz) and very low intensities (0.1–250 HT), which are non-invasive and safe for humans, were employed. CMFs can enhance cellular responses and interfere with microbial viability through physical stimulation, thus reducing the risk of toxicity and ecological impact. Specifically designed to modulate biological processes without thermal or cytotoxic effects, the CMFs represent a promising tool for antimicrobial therapy, particularly in the field of chronic wound healing.

The mentioned treatments were tested on the LCWB model and were evaluated 24 h after application. A recent study using in vitro models shows that, despite large initial reductions in biofilm viability (>99.9%), multi-species biofilms may begin to regrow within 24 h post-treatment [18]. Therefore, microbial viability checks should ideally be performed at 24 h to detect potential regrowth and assess the true efficacy of antimicrobial treatments. To explore the potential impact of strain variability and resistance profiles, two distinct polymicrobial mixes in the LCWB model were used, both containing *S. aureus*, *P. aeruginosa* and *C. albicans* obtained from different clinical samples.

Based on these considerations, the objective of this study was to evaluate the efficacy of MGO, LED and CMFs, applied alone and in combination with each other, on interkingdom biofilm in the LCWB model. After treatments, the biofilms were incubated for 24 h, and efficacy was assessed by evaluating the CFU/mg reduction as well as through detailed morphological and structural analyses using Scanning Electron Microscopy (SEM) and Confocal Laser Scanning Microscopy (CLSM).

## 2. Results

**Figure 1:**
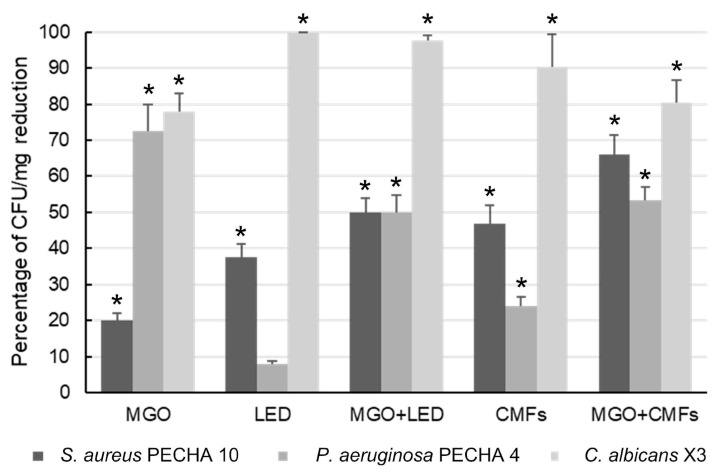
Percentages of CFU/mg reduction in *S. aureus* PECHA 10, *P. aeruginosa* PECHA 4 and *C. albicans* X3 (MIX 1) in LCWBs in presence of MGO, LED, CMFs and their combinations. Results were evaluated 24 h after treatments. * Statistically significant (*p*  <  0.05) value compared to the untreated controls. Results are expressed as mean ± standard deviation (SD) of four independent experiments, all performed on separate days. Error bars = SD. Abbreviations: LCWB—Lubbock Chronic Wound Biofilm; MGO—Methylglyoxal; LED—Light-Emitting Diode; CMFs—Complex Magnetic Fields.

Figure 1 shows the antibiofilm activity of different treatments against the MIX 1 triadic species (*S. aureus* PECHA 10, *P. aeruginosa* PECHA 4 and *C. albicans* X3) in the LCWB model. The antibiofilm action was evaluated 24 h after treatment, expressed as a percentage of CFU/mg biofilm reduction compared to the untreated control. All data, except LED treatment vs. *P. aeruginosa*, were statistically significant (*p*  <  0.05) in respect to the controls. MGO treatment induced a significant CFU/mg reduction of 77.83% ± 5.1 for *C. albicans*, 72.68% ± 7.3 for *P. aeruginosa* and 20.00% ± 2.2 for *S. aureus.* LED treatment reduced *C. albicans* and *S. aureus* by 98.90% ± 0.64 and 37.50% ± 3.6, respectively. The combination MGO + LED significantly enhanced the antibiofilm activity against *S. aureus* (49.85% ± 4.1) compared to both MGO (*p*  <  0.0001) and LED (*p*  =  0.007). Bliss independence analysis revealed an additive interaction. In addition, MGO + LED induced a significant reduction (*p*  <  0.0001) of 49.88% ± 4.9 of *P. aeruginosa* in respect to LED. CMFs induced a significant reduction of 90.34% ± 9.0 for *C. albicans*, of 47.00% ± 5.0 for *S. aureus* and of 24.13% ± 2.5 for *P. aeruginosa*, in respect to the controls. The combination MGO + CMFs showed a significant reduction in *S. aureus* (66.00% ± 5.5) compared to the single treatments (*p*  <  0.0001), and Bliss independence analysis confirmed a synergistic interaction. In addition, MGO + CMFs significantly reduced (*p*  <  0.0001) the count of *P. aeruginosa* (53.48% ± 3.7) in respect to CMFs.

**Figure 2:**
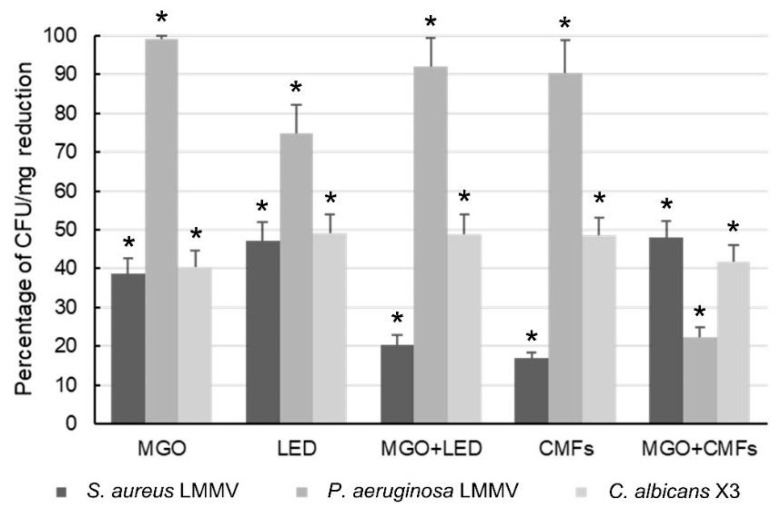
Percentages of CFU/mg reduction in *S. aureus* LMMV, *P. aeruginosa* LMMV and *C. albicans* X3 (MIX 2) in LCWBs in presence of MGO, LED, CMFs and their combinations. Results were evaluated 24 h after treatments. * Statistically significant (*p*  <  0.05) value compared to the untreated controls. Results are expressed as mean ± standard deviation (SD) of four independent experiments, all performed on separate days. Error bars = SD. Abbreviations: LCWB—Lubbock Chronic Wound Biofilm; MGO—Methylglyoxal; LED—Light-Emitting Diode; CMFs—Complex Magnetic Fields.

The results of the antibiofilm test against MIX 2 (*S. aureus* LMMV, *P. aeruginosa* LMMV and *C. albicans* X3) in the LCWB model are shown in Figure 2. The treatments caused a significant CFU/mg reduction in respect to the control, with variable antibiofilm efficacy against the three microorganisms of MIX 2. MGO caused the maximum reduction against *P. aeruginosa* (99.01% ± 0.9 CFU/mg reduction) and lower percentages of reduction against both *S. aureus* (38.68% ± 3.9) and *C. albicans* (40.54% ± 4.2). Similarly, LED treatment caused a high reduction in *P. aeruginosa* (74.94% ± 7.2) and induced comparable percentages of reduction against both *S. aureus* and *C. albicans* (47.11% ± 4.7 and 49.02% ± 5.05, respectively). The combination MGO + LED significantly enhanced (*p*  =  0.009) the effect of LED against *P. aeruginosa* (92.15% ± 7.3) and maintained the antifungal activity against *C. albicans* (49.02% ± 5.0) in respect to the single treatments. CMFs were highly effective against *P. aeruginosa* (90.27% ± 8.7 of CFU/mg reduction) and showed moderate antibiofilm efficacy against *C. albicans* (48.51% ± 4.7). The combination MGO + CMFs significantly enhanced the antibiofilm activity against *S. aureus* (47.94% ± 4.4) compared to both single treatments (*p* = 0.019 vs. MGO, *p* < 0.0001 vs. CMFs). Bliss independence analysis revealed an additive interaction.

**Figure 3:**
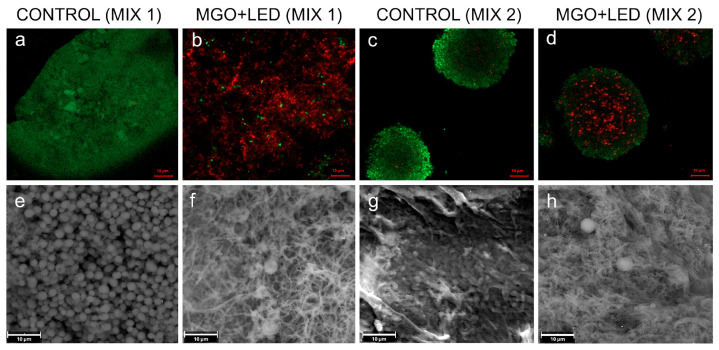
Representative CLSM and SEM images of LCWBs, comparing the effects of MGO + LED treatment on MIX 1 (*S. aureus* PECHA 10, *P. aeruginosa* PECHA 4, *C. albicans* X3) (**b**,**f**) and MIX 2 (*S. aureus* LMMV, *P. aeruginosa* LMMV, *C. albicans* X3) (**d**,**h**), in comparison to untreated controls (MIX 1: (**a**,**e**); MIX 2: (**c**,**g**)). CLSM observation (**a**–**d**): Original magnification 1000× (scale bars = 10 μm). SEM observation (**e**–**h**): Original magnification, 3600× (scale bars = 10 μm). Abbreviations: CLSM—Confocal Laser Scanning Microscopy; SEM—Scanning Electron Microscopy; LCWB—Lubbock Chronic Wound Biofilm; MGO—Methylglyoxal; LED—Light-Emitting Diode.

**Figure 4:**
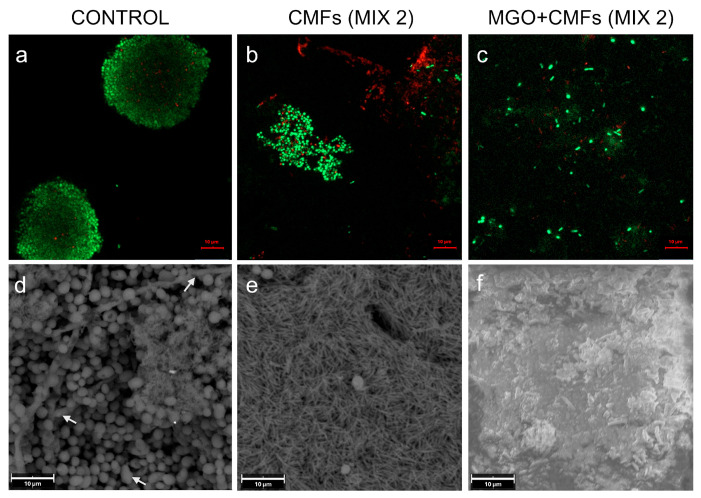
Representative CLSM and SEM images of LCWBs comparing the difference in effect between CMFs (**b**,**e**) and MGO + CMFs (**c**,**f**) treatments on MIX 2 (*S. aureus* LMMV, *P. aeruginosa* LMMV, *C. albicans* X3) in respect to the controls (**a**,**d**). CLSM observation (**a**–**c**): Original magnification 1000× (scale bars = 10 μm). SEM observation (**d**–**f**): Original magnification, 3600× (scale bars = 10 μm). White arrows (**d**) indicate pseudohyphae. Abbreviations: CLSM—Confocal Laser Scanning Microscopy; SEM—Scanning Electron Microscopy; LCWB—Lubbock Chronic Wound Biofilm; MGO—Methylglyoxal; CMFs—Complex Magnetic Fields.

Figure 3 and Figure 4 show representative images regarding the effect of the different treatments, evaluated after 24 h, obtained through CLSM after Live/Dead staining and SEM analysis. The CLSM images showed the capability of MGO + LED to disaggregate the biofilm and to induce a strong killing effect in MIX 1 and MIX 2 (Figure 3b,d). Regarding the effect on MIX 2, clusters of cells were still observed after CLSM analysis (Figure 3d), but a high percentage of dead cells was detected. Notably, the SEM observation (Figure 3f,h) revealed a near-complete eradication of *C. albicans* and a visible alteration of the fibrin network, compared to the untreated controls (Figure 3e,g).

Figure 4 shows CLSM and SEM images of CMFs and MGO + CMFs, evaluated 24 h after treatment, against the microorganisms of MIX 2 in the LCWB model. In untreated samples, clusters of viable bacterial cells were detected by CLSM (Figure 4a). SEM analysis revealed the cluster organization of *C. albicans* cells with adherent *P. aeruginosa* and *S. aureus* (Figure 4d), as well as the presence of pseudohyphae, indicated by white arrows. Figure 4b,e shows the effect of CMFs against MIX 2, evaluated 24 h post-treatment, resulting in a microbial cluster disaggregation and a reduction in *P. aeruginosa* cell viability, confirmed by CLSM analysis. The MGO + CMFs (Figure 4c) combination enhanced cluster disaggregation and significantly reduced cell viability compared to CMFs alone (Figure 4b). SEM analysis showed the capability of CMFs to inhibit *Candida* yeast-to-hyphae transition (Figure 4e). Moreover, the analysis in Figure 4f shows that MGO + CMFs also exerted its antimicrobial effect through a wrapping mechanism, entrapping microbial cells and limiting their viability.

**Figure 5:**
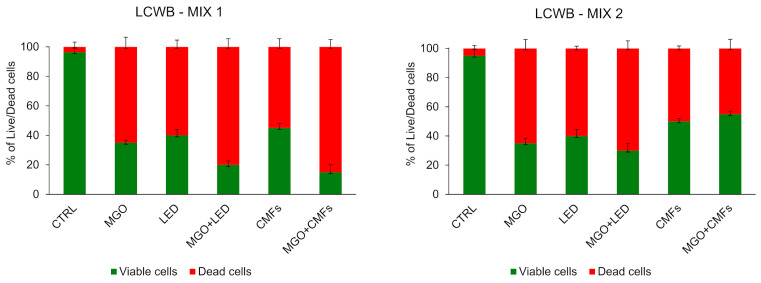
Percentages of viable/dead cells of the microbial population in LCWB–MIX 1 and LCWB–MIX 2 after the treatments, compared with the untreated control (CTRL). The samples were observed with CLSM using a Live/Dead staining BacLight viability kit. (Error bars = standard deviation). Abbreviations: LCWB—Lubbock Chronic Wound Biofilm; MGO—Methylglyoxal; LED—Light-Emitting Diode; CMFs—Complex Magnetic Fields; CLSM—Confocal Laser Scanning Microscopy.

Figure 5 represents the viable/dead distribution of the microbial community in the LCWBs for all applied treatments. Variable reductions can be noticed in all conditions, with the majority of red cells coming from when LCWB-MIX 1 was treated with the combinations MGO + LED (80% of dead cells) or MGO + CMFs (85% of dead cells).

## 3. Discussion

Antimicrobial resistance represents a serious issue in the healthcare system due to the ability of microorganisms to adapt to the treatments, leading to the failure of common antimicrobial therapies. The search for alternative non-antibiotic approaches is a fundamental step in the management of microbial colonization in chronic wounds [19].

In this context, the present research investigated the antibiofilm efficacy of MGO, LED and CMFs, alone and in combination, against an in vitro Lubbock Chronic Wound Biofilm (LCWB) model, evaluated 24 h after treatments. These strategies have previously demonstrated significant antimicrobial efficacy against both planktonic cells and biofilm-embedded microorganisms and may represent a promising component of effective protocols in chronic wound management [20,21]. The ideal treatment should promote antibacterial effects while minimizing cytotoxic effects. Several studies showed that MGO affects cell viability, proliferation, and apoptosis at high concentrations [22,23]. Therefore, at the concentration used in this study, MGO is non-toxic and safe for mammalian cells.

Regarding the experimental part, the LCWB model was prepared using two different polymicrobial combinations: MIX 1 (*S. aureus* PECHA 10, *P. aeruginosa* PECHA 4, *C. albicans* X3) and MIX 2 (*S. aureus* LMMV, *P. aeruginosa* LMMV, *C. albicans* X3). These mixes included strains with known resistance profiles [20,24] and were chosen to reflect different clinical scenarios of chronic wound infection. Then, studying the effects of different strategies on both microbial mixes 24 h after treatments allowed a comparative evaluation of the efficacy of the proposed therapies against two distinct, resistance-associated biofilm models.

The obtained results confirmed that all tested treatments showed significant antibiofilm activity, compared to controls, against the microbial components within the LCWBs. Diban et al. used the same therapies against the planktonic phase of microbial strains isolated from chronic wounds tested separately, demonstrating significant antimicrobial activity [20]. Instead, the results of the present study reflect the effect on biofilm microbial strains 24 h post-treatment and confirm that the reduction in the viable cell count is similar to the previous results, with a major reduction in *Candida* and a lower effect on the bacterial strains. In this study, based on data reported by Diban et al. [20], MGO was tested at a concentration of 32 µg/mL (2xMIC) and demonstrated a significant antibiofilm activity within both triadic-species LCWB models (MIX 1 and MIX 2), despite the increased tolerance associated with biofilm formation [25]. The remarkable antibiofilm effect could be attributed to the antimicrobial activity of MGO during 24 h of application, which allowed the compound to better act on the microbial strains embedded within the biofilm structure.

Although MGO alone showed significant efficacy, the primary objective of the present study was to combine it with low-environmental-impact technologies, represented by LED and CMFs, to potentiate their antibiofilm activity.

The CFU/mg biofilm reductions varied among the tested microbial strains (MIX 1, MIX 2) and the applied treatments. Among the species in MIX 1, *C. albicans* exhibited the highest susceptibility to all treatments in terms of CFU/mg reduction, as also confirmed by microscopic observations. Notably, red LEDs (630 nm), alone and in combination with MGO, caused a significant reduction in the fungal cell count, compared to unexposed cells. This result is in line with previous studies that have explored photoinactivation mediated by other photosensitizers combined with red LED against *C. albicans* biofilms. D’Amico et al. applied 5% delta aminolaevulinic acid combined with 630 nm LED against a clinically resistant *C. albicans* strain, and they observed reductions in fungal biofilm biomass by 97.10% without any cytotoxic effects on human gingival fibroblast [16]. De Oliveira Mima et al. demonstrated that LED combined with Photogem, a hematoporphyrin derivative, significantly reduced the viability of *C. albicans* biofilm in vivo without harming the oral mucosal tissue [26]. These findings suggest that photoinactivation caused by LED can induce oxidative stress following high levels of reactive oxygen species (ROS) production that leads to irreversible microbial cell damage [16,27,28].

In MIX 2, *P. aeruginosa* was the most susceptible strain, especially following treatment with MGO, LED, CMFs and MGO + LED.

These observations suggest that the composition of the microbial community and the potential interspecies interactions can influence treatment outcomes. This observation aligns with previous studies demonstrating that biofilm-forming capacity and antimicrobial tolerance are highly dependent on strain-specific variability [9,25].

The MGO + CMFs combination, 24 h after treatment, induced a significant CFU/mg biofilm reduction and demonstrated a marked capability to disaggregate biofilms. This combination significantly increased antibiofilm activity against *S. aureus* in both polymicrobial mixes compared to MGO and CMFs alone, with a synergistic interaction observed in *S. aureus* PECHA 10, and improved the antibiofilm activity against *P. aeruginosa* in MIX 1 compared to CMFs alone. A potential mechanism of action of MGO + CMFs has been identified by Diban et al. in a study that showed the capability of this combination to affect membrane fluidity and permeability in the planktonic phase of *S. aureus* and *P. aeruginosa* [20]. A reduction in membrane fluidity, along with changes in membrane fatty acid composition, is known to influence microbial adhesion [29] and may increase membrane permeability, making bacterial cells more susceptible to the cytotoxic effect of the combined treatment. Another important aspect is the capacity of CMFs to inhibit the yeast-to-hyphae transition of *C. albicans*, as observed by SEM, since filamentation is crucial for tissue invasion and biofilm development [4]. Di Lodovico et al. demonstrated the efficacy of CMFs in inhibiting growth and promoting the disaggregation of biofilms formed by a resistant *C. albicans* strain, associated with no morphological *C. albicans* changes in respect to the untreated control and the absence of cytotoxic effects on human gingival fibroblasts [21], further supporting the potential of CMFs as a promising therapy for disrupting fungal biofilms.

The combination MGO + LED, 24 h after treatment, enhanced the antibiofilm effect compared to LED against *P. aeruginosa* (in both MIX 1 and MIX 2) and *S. aureus* (MIX 1). Additionally, this combination potentiated the activity of MGO against *S. aureus* (MIX 1) and *C. albicans* (MIX 1 and MIX 2). The interaction between the two antimicrobial agents is possibly due to the multi-targeted activity of both treatments, in which MGO acts on the microbial cell wall, DNA and efflux pump system, while LED can increase the oxidative stress [16,30,31].

On the other hand, in the case of polymicrobial co-infection, the complex interactions between microorganisms may influence the overall response to the treatment by altering the susceptibility and survival of individual species. A difference in microbial viability between the two mixes after the combined treatment was observed with CLSM after 24 h of treatment, with a higher number of dead cells in LCWB-MIX 1 in comparison to LCWB-MIX 2. However, microscopic observations also suggest that MGO + LED and MGO + CMFs can alter the biofilm structure, with disaggregating action compared to the control, suggesting the ability of the applied combinations to influence the biofilm network as a possible mechanism of action. Therefore, a reduced biofilm formation ability will result in the production of subtle fibrils that negatively affect microbial accumulation in the three-dimensional structure of the biofilm.

Several studies have shown that even after significant biofilm reduction, microbial regrowth can occur within 24–48 h if it is not completely eradicated [17,32]. This supports the hypothesis that biofilm resilience against antimicrobial treatments is not only determined by microbial viability but also by the integrity of the protective extracellular polymeric matrix, which acts as a reservoir for microbial recolonization [33].

These findings underscore the importance of targeting not only microbial cells but also the biofilm matrix and the species interactions that support biofilm resilience. Polymicrobial biofilms are dynamic ecosystems where bacteria and/or fungi cooperate to resist environmental stresses, such as antimicrobial treatments.

This study demonstrates that non-antibiotic combination therapies can effectively disrupt this equilibrium.

The light transmission through tissue is considered an important factor in the actual application of this method. According to Barolet’s study, red LEDs (630 nm) have the deepest penetration among the visible light wavelengths, with a penetration depth of 2 mm [34]. The thickness of the LCWB model is within this range, making it a suitable model for studying dermal tissue treatments, including photodynamic therapy. Also, red LED (630 nm) application on human fibroblasts showed a high safety profile, increasing cell proliferation and migration, suggesting its promising role in wound healing applications [16,35,36]. LED therapy could therefore represent a valid method to apply to wound infections due to its ability to enhance the wound healing process along with the antimicrobial action against wound pathogens.

Regarding the ability of CMFs to influence dermal tissues, the evidence reported in De Santis’s study proposes that complex electromagnetic fields can penetrate skin thickness, reaching the dermis [37]. The CMF device used in this study was also applied by Zanotti et al., who demonstrated the safety of CMFs on human fibroblasts by performing the MTT assay associated with the absence of side effects on cell physiology [38]. The previous literature demonstrates a remarkable effect of complex electromagnetic fields, not only as an antimicrobial method, but also as a suggested technique that improves the wound healing process [21,38].

Future studies should be conducted to highlight other possible mechanisms of action.

A limit of this study is that the LCWB model does not fully replicate the complex host–pathogen and inflammatory interactions that occur in vivo for the absence of an immune system component. Moreover, although the results are promising, these assays underline the importance of monitoring remaining adherent cells due to the risk of selection of resistant populations.

## 4. Materials and Methods

### 4.1. Methylglyoxal (MGO) Aqueous Dispersion

Pyruvaldehyde solution (Methylglyoxal, MGO) at 40 wt.% in H_2_O (Sigma-Aldrich, Milan, Italy) was diluted in sterile Phosphate-Buffered Saline (PBS; Merck KGaA, Darmstadt, Germany) to obtain a final concentration of 32 µg/mL. This concentration was chosen based on data reported by Diban et al. [20], where 16 µg/mL MGO was identified as effective against the most resistant microbial strain when applied in combination with LED or CMFs, showing synergistic antimicrobial effects. For experiments performed on the LCWB model, the MGO concentration was increased twofold from 16 µg/mL to 32 µg/mL. The MGO application time was 22 min, similar to the application time of CMFs.

### 4.2. Light-Emitting Diode (LED) Device

A light-emitting diode (LED) device (TL-06; Alpha Strumenti, Melzo, Milan, Italy) emitting at a wavelength of 630 nm ± 10 nm was employed to irradiate the LCWB samples for 17 min, following the protocol described by Di Lodovico et al. [15]. The irradiation was delivered through a handpiece equipped with 12 LEDs arranged in a 3 × 4 rectangular configuration, providing a site-specific irradiation power of 30 mW. The energy density is calculated as following: J/cm^2^ = (mW/cm^2^)/1000 × time(s) = (30/1000) × 1020 = 30.6 J/cm^2^.

### 4.3. Complex Magnetic Fields Source (CMFs)

The CMF device (Next sx version, Medicina Fisica Integrata, M.F.I., Rome, Italy) was applied to LCWB samples for 22 min using the STRESS program, which is characterized by multifrequency magnetic fields of variable frequency, intensity, duration, and waveform [21]. This device emits pulsed electromagnetic fields with intensities ranging from 0.1 to 250 µT and frequencies between 1 and 250 Hz. The magnetic field is generated through coils consisting of 650 turns of 0.35 mm wide enameled copper wire. The external dimensions of the coil are 110 mm, with a 12 mm internal diameter and 8 mm thickness.

### 4.4. Microbial Cultures

Clinical isolates *S. aureus* PECHA 10, *S. aureus* LMMV, *P. aeruginosa* PECHA 4, *P. aeruginosa* LMMV and *C. albicans* X3 were selected from the private strain collection of the Bacteriology Laboratories at the Department of Pharmacy, University “G. d’Annunzio” of Chieti and used for the experiments [15,20]. These bacteria were isolated from patients with chronic wounds who had provided informed consent and were cultured on selective media: Mannitol Salt Agar (MSA, Oxoid, Milan, Italy) for *S. aureus*, Cetrimide Agar (CET, Oxoid, Milan, Italy) for *P. aeruginosa* and Sabouraud Agar (SAB, Oxoid, Milan, Italy) for *C. albicans*. Bacteria pure colonies were inoculated in Trypticase Soy Broth (TSB, Oxoid) and incubated overnight at 37 °C in aerobic conditions, then refreshed for 2 h at 37 °C in an orbital shaker. The bacterial suspensions were adjusted to an optical density (OD_600_) of 0.125 using a Biophotometer (Eppendorf, Milan, Italy) and subsequently diluted 1:10 for *S. aureus* and 1:100 for *P. aeruginosa*, to obtain final concentrations of 10^6^ and 10^5^ colony-forming units per milliliter (CFU/mL), respectively. For *C. albicans* X3, colonies grown on SAB agar were used to prepare a broth culture in TSB adjusted to an OD_600_ of 0.15, corresponding to approximately 5 × 10^5^ CFU/mL [13].

### 4.5. Lubbock Chronic Wound Biofilm (LCWB) Model Assay

The LCWB (Lubbock Chronic Wound Biofilm) model was prepared following a previously described methodology [13]. Four independent experiments were performed using the LCWB model, all conducted on separate days. For each experiment, one Lubbock sample was prepared for each tested condition (control group and treated groups). Inter-sample variation was controlled by preparing Lubbock samples with identical composition and under identical experimental conditions. The LCWB model contained 5 mL of Brucella Broth (BB, Oxoid, Milan, Italy) with 0.1% bacteriological agar, 50% porcine plasma (Sigma-Aldrich, Milan, Italy), 5% horse erythrocytes (BBL Microbiology Systems, Milan, Italy) and 2% fetal calf serum (Biolife Italiana, Milan, Italy). This medium was dispensed into sterile glass tubes. Two distinct polymicrobial inocula, each containing specific strains of *S. aureus*, *P. aeruginosa*, and *C. albicans*, were prepared as follows:

- MIX 1: *S. aureus* PECHA 10, *P. aeruginosa* PECHA 4 and *C. albicans* X3.
- MIX 2: *S. aureus* LMMV, *P. aeruginosa* LMMV and *C. albicans* X3.

Five microliters of each diluted broth culture were inoculated into the glass tubes with a sterile pipette tip. The glass tubes were incubated aerobically for 48 h at 37 °C. After incubation, the pipette tips were removed, and the biofilm biomass was gently washed twice with sterile PBS. The biomass was then weighed (in mg), and the biovolume was calculated using the formula: V = π × r^2^ × h. The LCWBs were then transferred onto an artificial “wound bed” created using oval-shaped sterile 1.5 mL Eppendorf tubes positioned on an agar-based nutrient medium prepared with Bolton Broth (Oxoid, Milan, Italy) containing 1.5% agar.

### 4.6. Effect of MGO, LED and CMFs Alone and in Combination on LCWB Model

After placing the mature biofilm on the artificial “wound bed”, LCWBs were treated with MGO, LED, CMFs, MGO + LED and MGO + CMFs. The volume of MGO (at a final concentration of 32 μg/mL) or PBS (used as an untreated control) depended on each LCWB volume. The volume was determined to prevent the diffusion of the substances into the Bolton medium wound bed and to ensure complete adsorption only on the LCWB surface. The application times were 22 min for MGO, 17 min for LED and 22 min for CMFs. The combined effects of MGO and LED/CMFs were evaluated by first applying MGO and simultaneously applying LED or CMFs treatment for the corresponding exposure time (17 min for LED and 22 min for CMFs). After the treatment, untreated and treated LCWBs were incubated at 37 °C for 24 h. Then, each Lubbock (treated and untreated) was removed from the artificial “wound bed”, vortexed for 2 min in 1 mL of PBS, sonicated for 3 min with an ultrasound bath (Elmasonic P60H; Elma Schmidbauer GmbH, Singen, Germany), and vortexed for another 2 min. The resulting suspension was serially diluted in PBS and plated in triplicate to determine the number of colony-forming units (CFU/mL). Differential media were used to quantify each microbial species composing the model: MSA for *S. aureus*, CET for *P. aeruginosa* and SAB for *C. albicans*. Plates were incubated at 37 °C for 24–48 h. After incubation, colonies were counted and the mean of the CFU/mL value obtained for each species and each sample was divided by the corresponding Lubbock weight (mg) to express the results as CFU/mg, in order to normalize biomass differences. The percentage of CFU/mg reduction in respect to the control was calculated.

Results were expressed as mean ± SD of the percentage of CFU/mg reduction in each species obtained from the four independent experiments. The source data file containing CFU counts per mg per replicate is provided as Supplementary Material Table S2.

The calculations to determine the synergy based on Bliss’s definition of drug independence were performed [39].

### 4.7. Scanning Electron Microscopy (SEM) Analysis

For Scanning Electron Microscopy (SEM) examination, untreated or treated LCWB samples were washed with PBS, fixed in 2.5% glutaraldehyde, washed three times with PBS, dehydrated using scalar solutions at increasing doses of ethanol and then immersed in hexamethyldisilazane (HMDS, Sigma-Aldrich, Milan, Italy). HMDS was decanted from the specimen vial and the tissues were left to air dry at room temperature. The dried samples were subjected to gold-sputtering with a Desk Sputter Coater (Phenom-World B.V., Dillenburgstraat 97, Eindhoven, 5652, AM, The Netherlands) and then observed with SEM (Phenom-World B.V., Dillenburgstraat 97, Eindhoven, 5652, AM, The Netherlands) at different magnifications.

### 4.8. Confocal Laser Scanning Microscopy (CLSM) Analysis

The untreated and treated LCWB samples were also analyzed by CLSM using a Live/Dead staining BacLight viability kit (Invitrogen, Milan, Italy). The LCWB samples were washed in sterile PBS in order to remove the non-adhered bacteria and subsequently stained with a BacLight kit (Thermo Fisher Scientific, Waltham, MA, USA) for 15 min in the dark, at room temperature. Samples were then examined using a Zeiss LSM800 microscope (Carl Zeiss, Jena, Germany) coupled to an inverted microscope Axio-observer D1 (Carl Zeiss, Jena, Germany) equipped with a Plan Neofluaroil-immersion objective (100×/1.45 NA). The green and red emission (SYTO 9 and propidium iodide, respectively) were excited using the 488 nm setting (4% of potency) of an argon laser and a helium/neon 543 nm source (2.5% of potency). To separate the fluorescence emissions, HTF 488/543 and NTF 545 as primary and secondary dichroic mirrors, respectively, were used. Detector band-pass filters were set over 505–530 and 565–615 ranges for the green and red emissions, respectively. Images were alternatively recorded using the multitrack acquisition [40]. For the evaluation of microbial viability, five random fields for each group were observed, and the mean values (+/− standard deviation) were considered for the statistical analysis.

### 4.9. Statistical Analysis

All data were obtained from four independent experiments performed in triplicate. Data are shown as the mean value ± standard deviation (SD) of four independent experiments. Statistical significance was determined using SPSS for Windows version 21 (IBM SPSS Inc., Chicago, IL, USA). Differences between treatments for each microorganism were assessed with one-way analysis of variance (ANOVA) with Tukey post hoc test (Supplementary Material-Tables S1 and S2). *p*-values ≤ 0.05 were taken as being statistically significant.

## 5. Conclusions

This study demonstrates that MGO alone and combined with LED or CMFs can significantly affect resistant strains embedded in the interkingdom biofilm in the LCWB model 24 h after treatments. The use of these antimicrobial approaches is supported by several studies regarding their biocompatibility and lack of toxicity toward human cells under the conditions used in this study. Future studies are needed before these methods can be used in clinical practice, but the results presented certainly encourage and support the use of non-antibiotic, eco-sustainable combination strategies as alternative, non-invasive therapeutic approaches to conventional antibiotic therapy in the management of chronic wound infections.

## Data Availability

The original contributions presented in this study are included in the article/Supplementary Materials. Further inquiries can be directed to the corresponding author. The data presented in this study are openly available in the article and as Supplementary Materials.

## Supplementary Materials

The following supporting information can be downloaded at: https://www.mdpi.com/article/10.3390/ijms262311658/s1.

## Abbreviations

The following abbreviations are used in this manuscript:

CFU/mg	Colony-Forming Unit per milligram
CLSM	Confocal Laser Scanning Microscopy
CMFs	Complex Electromagnetic Fields
EPS	Extracellular Polymeric Substances
LCWB	Lubbock Chronic Wound Biofilm
LED	Light-Emitting Diodes
MGO	Methylglyoxal
SEM	Scanning Electron Microscopy
